# (4*S*,5*R*)-4-Benz­yloxy-5-[4-(cyclo­hexa­ne­carbon­yl)phen­yl]-1-(4-meth­oxy­benz­yl)pyrrolidin-2-one

**DOI:** 10.1107/S1600536814003638

**Published:** 2014-03-12

**Authors:** Yan-Jiao Gao, Jie Ma, Xiao Zheng

**Affiliations:** a The Key Laboratory for Chemical Biology of Fujian Province, College of Chemistry and Chemical Engineering, Xiamen University, Xiamen, Fujian 361005, People’s Republic of China

## Abstract

The title compound, C_32_H_35_NO_4_, is an unexpected product obtained in the SmI_2_-mediated radical cross-coupling of a lactam 2-pyridyl sulfone with an arone. The asymmetric unit contains two mol­ecules. In both mol­ecules, the core pyrrolidinone ring adopts an approximate envelope conformation (with the C atom bearling the benzyloxy substituent as the flap) and the cyclo­hexyl ring has a chair conformation. The relative orientation of the two substitutent groups at the 4- and 5-positions of the pyrrolidinone ring is *anti* in both mol­ecules, with O(benz­yloxy)—C—C—C(benzene) torsion angles of 150.8 (3) and 154.2 (2)°. In the crystal, C—H⋯O inter­actions involving carbonyl groups as acceptors lead to the formation of a tape motif propagating parallel to the *a-*axis direction.

## Related literature   

For backround to the synthesis, see: Shiue *et al.* (1997 ▶); Zheng *et al.* (2005 ▶); Hu *et al.* (2013 ▶).
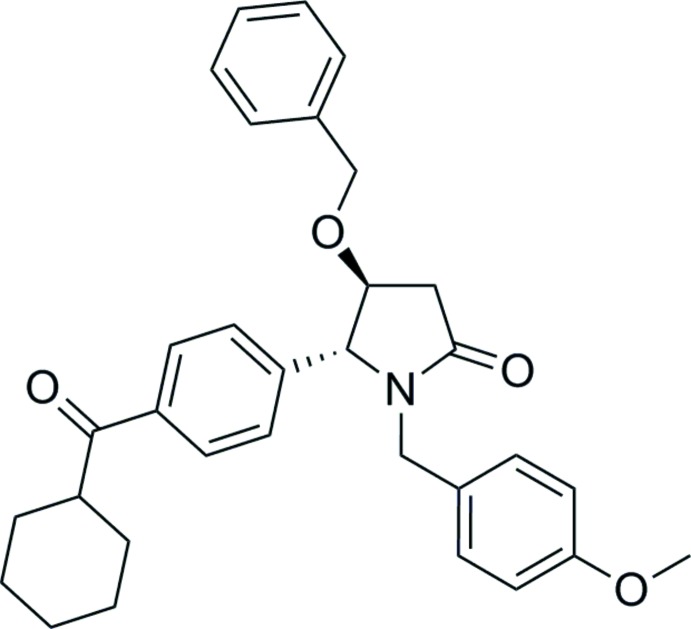


## Experimental   

### 

#### Crystal data  

C_32_H_35_NO_4_
*M* *_r_* = 497.61
Monoclinic, 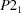

*a* = 9.4964 (4) Å
*b* = 28.6497 (13) Å
*c* = 9.9511 (4) Å
β = 96.727 (4)°
*V* = 2688.8 (2) Å^3^
*Z* = 4
Mo *K*α radiation
μ = 0.08 mm^−1^
*T* = 100 K
0.2 × 0.12 × 0.09 mm

#### Data collection  

Oxford Diffraction SuperNova diffractometer
Absorption correction: multi-scan (*CrysAlis PRO*; Oxford Diffraction, 2006 ▶) *T*_min_ = 0.927, *T*_max_ = 1.000
11184 measured reflections
7540 independent reflections
6140 reflections with *I* > 2σ(*I*)
*R*_int_ = 0.037

#### Refinement  

*R*[*F* ^2^ > 2σ(*F* ^2^)] = 0.048
*wR*(*F* ^2^) = 0.123
*S* = 1.03
7540 reflections
669 parameters
1 restraint
H-atom parameters constrained
Δρ_max_ = 0.21 e Å^−3^
Δρ_min_ = −0.18 e Å^−3^

### 

Data collection: *CrysAlis PRO* (Oxford Diffraction, 2006 ▶); cell refinement: *CrysAlis PRO*; data reduction: *CrysAlis PRO*; program(s) used to solve structure: *SHELXTL* (Sheldrick, 2008 ▶); program(s) used to refine structure: *SHELXTL*; molecular graphics: *SHELXTL*; software used to prepare material for publication: *SHELXTL*.

## Supplementary Material

Crystal structure: contains datablock(s) I. DOI: 10.1107/S1600536814003638/fy2104sup1.cif

Structure factors: contains datablock(s) I. DOI: 10.1107/S1600536814003638/fy2104Isup2.hkl

Supporting information file. DOI: 10.1107/S1600536814003638/fy2104Isup3.cdx

Supporting information file. DOI: 10.1107/S1600536814003638/fy2104Isup4.cml

CCDC reference: 987472

Additional supporting information:  crystallographic information; 3D view; checkCIF report

## Figures and Tables

**Table 1:** Hydrogen-bond geometry (Å, °)

*D*—H⋯*A*	*D*—H	H⋯*A*	*D*⋯*A*	*D*—H⋯*A*
C62—H62⋯O4^i^	0.93	2.47	3.260 (5)	143
C17—H17⋯O2^ii^	0.93	2.38	3.237 (4)	153
C49—H49⋯O6^ii^	0.93	2.33	3.190 (4)	154

Symmetry codes: (i) 
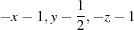
; (ii) 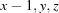.

**Fig. 1.**
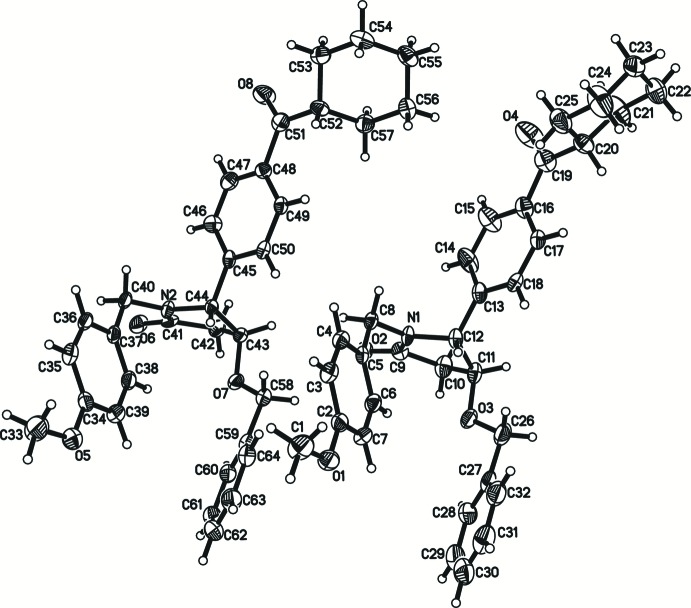
The molecular structure of the title compound showing 50% probability displacement ellipsoids.

**Fig. 2.**
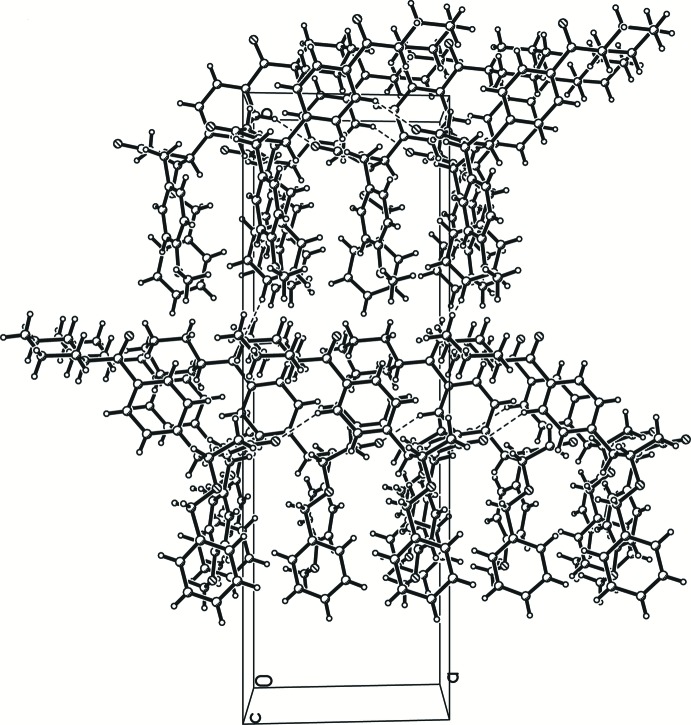
The packed unit cell.

## supplementary crystallographic information

### 1. Comment

In the SmI_2_/HMPA-promoted phenyl-carbonyl coupling reaction, a special type of phenyl radicals were generated from benzaldehyde and acetophenones by Shiue *et al.* (1997). In addition, using SmI_2_ as the single electron reductant, acylaminoalkyl radicals were generated from 2-pyridyl sulfides or 2-pyridyl sulfones (Zheng *et al.*, 2005; Hu *et al.*, 2013). So, the SmI_2_-mediated phenyl-pyrrolidyl coupling of (4*S*)-1-(4-methoxybenzyl)-4-benzyloxy-5-(pyridin-2-ylsulfonyl)pyrrolidin-2-one with benzoylcyclohexane produced (4*S*,5*R*)-4-(benzyloxy)-5-(4-(cyclohexanecarbonyl)phenyl)-1-(4-methoxybenzyl)pyrrolidin-2-one reasonably. Here we report the structure of the title compound.

### 2. Experimental

To a solution of (4*S*)-1-(4-methoxybenzyl)-4-benzyloxy-5-(pyridin-2-ylsulfonyl)pyrrolidin-2-one (0.5 mmol) and benzoylcyclohexane (1.5 mmol) in dry THF (10 ml) was added a freshly prepared *t*-BuOH-containing SmI_2_ (0.1 *M* in THF, 20 ml, 2.0 mmol) at -60 °C. After being stirred for 2 h, the reaction was quenched with a saturated aqueous solution of NH_4_Cl (10 ml), and the resulting mixture was extracted with EtOAc (3 × 15 ml). The combined organic layers were washed with brine, dried over anhydrous Na_2_SO_4_, filtered and concentrated under reduced pressure. The residue was purified by flash chromatography on silica gel (eluent: EtOAc/Hex = 1: 10) to afford the title compound (white crystals, yield 35%). Single crystals of the tiltle compound were obtained by slow evaporation of a mixture of *n*-hexane/dichloromethane solution. The title compound was prepared from an optical pure starting material and no racemization was observed in this reaction.

IR (film): 3066, 3027, 2928, 1716, 1611, 1513, 1248, 1316, 1108 cm^-1^; ^1^H NMR (400 MHz, CDCl_3_) δ 1.3–2.0(m,10*H*), 2.59(dd, *J* = 3.1, 17.5 Hz,1*H*), 2.85(dd, *J* = 6.8, 17.5 Hz,1*H*), 3.24(m, 1H), 3.49 (d, *J* = 14.8 Hz, 1H), 3.78(s, 3H), 3.94 (ddd appare. dt, *J* = 2.4, 3.1, 6.8 Hz, 1H), 4.44 (d, *J* = 12.1 Hz, 1H), 4.47 (d, *J* = 12.1 Hz, 1H), 4.49 (d, *J* = 2.4 Hz, 1H), 5.14 (d, *J* = 14.8 Hz, 1H), 6.80 (d, *J* = 8.7 Hz, 2H), 7.02 (d, *J* = 8.7 Hz, 2H), 7.14–7.20 (m,4*H*), 7.24–7.31 (m,3*H*), 7.91–7.96 (m,2*H*). ^13^C NMR (100 MHz, CDCl_3_) δ 25.8 (2 C), 25.9 (2 C), 29.4, 37.2, 43.8, 45.7, 55.3, 67.2, 71.3, 79.2, 114.1, 126.9, 127.6, 128.0, 128.5, 129.2, 129.6, 136.4, 137.2, 142.9, 159.1, 172.9, 203.2. HRESIMS calcd for [C_32_H_35_NO_4_Na]^+^ (*M* + Na^+^): 520.2464; found: 520.2474.

### 3. Refinement

The hydrogen atoms were positioned geometrically, with C—H = 0.93, 0.98, 0.97 and 0.96 Å for phenyl, methine, methylene and methyl H atoms, respectively, and were included in the refinement in the riding model approximation. The displacement parameters of methyl H atoms were set to 1.5 *U*_eq_(C), while those of other H atoms were set to 1.2 *U*_eq_(C). In the absence of significant anomalous scattering effects, Friedel pairs were merged. The absolute configuration was assigned with reference to the starting materials in the synthetic procedure.

### Crystal data

**Table:**

C_32_H_35_NO_4_	*Z* = 4
*M**_r_* = 497.61	*F*(000) = 1064
Monoclinic, *P*2_1_	*D*_x_ = 1.229 Mg m^−^^3^
*a* = 9.4964 (4) Å	Mo *K*α radiation, λ = 0.71073 Å
*b* = 28.6497 (13) Å	µ = 0.08 mm^−^^1^
*c* = 9.9511 (4) Å	*T* = 100 K
β = 96.727 (4)°	Columnar, colorless
*V* = 2688.8 (2) Å^3^	0.2 × 0.12 × 0.09 mm

### Data collection

**Table:**

Oxford Diffraction SuperNova diffractometer	7540 independent reflections
Radiation source: fine-focus sealed tube	6140 reflections with *I* > 2σ(*I*)
Graphite monochromator	*R*_int_ = 0.037
Detector resolution: 10.3415 pixels mm^-1^	θ_max_ = 25.0°, θ_min_ = 3.5°
ω scans	*h* = −11→9
Absorption correction: multi-scan (*CrysAlis PRO*; Oxford Diffraction, 2006)	*k* = −34→28
*T*_min_ = 0.927, *T*_max_ = 1.000	*l* = −11→11
11184 measured reflections	

### Refinement

**Table:**

Refinement on *F*^2^	Primary atom site location: structure-invariant direct methods
Least-squares matrix: full	Secondary atom site location: difference Fourier map
*R*[*F*^2^ > 2σ(*F*^2^)] = 0.048	Hydrogen site location: inferred from neighbouring sites
*wR*(*F*^2^) = 0.123	H-atom parameters constrained
*S* = 1.03	*w* = 1/[σ^2^(*F*_o_^2^) + (0.059*P*)^2^] where *P* = (*F*_o_^2^ + 2*F*_c_^2^)/3
7540 reflections	(Δ/σ)_max_ < 0.001
669 parameters	Δρ_max_ = 0.21 e Å^−^^3^
1 restraint	Δρ_min_ = −0.18 e Å^−^^3^

### Special details

**Table:**

Geometry. All esds (except the esd in the dihedral angle between two l.s. planes) are estimated using the full covariance matrix. The cell esds are taken into account individually in the estimation of esds in distances, angles and torsion angles; correlations between esds in cell parameters are only used when they are defined by crystal symmetry. An approximate (isotropic) treatment of cell esds is used for estimating esds involving l.s. planes.
Refinement. Refinement of *F*^2^ against ALL reflections. The weighted *R*-factor *wR* and goodness of fit *S* are based on *F*^2^, conventional *R*-factors *R* are based on *F*, with *F* set to zero for negative *F*^2^. The threshold expression of *F*^2^ > σ(*F*^2^) is used only for calculating *R*-factors(gt) *etc*. and is not relevant to the choice of reflections for refinement. *R*-factors based on *F*^2^ are statistically about twice as large as those based on *F*, and *R*- factors based on ALL data will be even larger.

### Fractional atomic coordinates and isotropic or equivalent isotropic displacement parameters (Å^2^)

**Table:**

	*x*	*y*	*z*	*U*_iso_*/*U*_eq_	
C1	0.2734 (5)	0.20483 (14)	0.1362 (4)	0.0441 (10)	
H1A	0.3236	0.2201	0.0703	0.066*	
H1B	0.1749	0.2132	0.1212	0.066*	
H1C	0.2827	0.1716	0.1279	0.066*	
C2	0.3430 (4)	0.26598 (12)	0.2923 (3)	0.0269 (8)	
C3	0.2965 (4)	0.30055 (12)	0.1985 (3)	0.0283 (8)	
H3	0.2514	0.2925	0.1136	0.034*	
C4	0.3183 (3)	0.34722 (12)	0.2335 (3)	0.0252 (8)	
H4	0.2871	0.3701	0.1707	0.030*	
C5	0.3848 (3)	0.36064 (11)	0.3586 (3)	0.0205 (7)	
C6	0.4272 (3)	0.32517 (12)	0.4524 (3)	0.0263 (8)	
H6	0.4710	0.3331	0.5378	0.032*	
C7	0.4050 (4)	0.27891 (12)	0.4197 (3)	0.0294 (9)	
H7	0.4319	0.2560	0.4840	0.035*	
C8	0.4129 (3)	0.41149 (11)	0.3902 (3)	0.0219 (7)	
H8A	0.5000	0.4208	0.3555	0.026*	
H8B	0.3363	0.4300	0.3444	0.026*	
C9	0.5492 (3)	0.43143 (11)	0.6091 (3)	0.0251 (8)	
C10	0.5213 (4)	0.43434 (14)	0.7556 (3)	0.0338 (9)	
H10A	0.5156	0.4666	0.7841	0.041*	
H10B	0.5959	0.4188	0.8141	0.041*	
C11	0.3807 (4)	0.40977 (13)	0.7596 (3)	0.0283 (8)	
H11	0.3262	0.4232	0.8278	0.034*	
C12	0.3050 (3)	0.41663 (12)	0.6133 (3)	0.0253 (8)	
H12	0.2521	0.3882	0.5851	0.030*	
C13	0.2059 (3)	0.45772 (12)	0.5976 (3)	0.0249 (8)	
C14	0.2547 (4)	0.50246 (13)	0.5807 (4)	0.0435 (10)	
H14	0.3512	0.5075	0.5788	0.052*	
C15	0.1629 (4)	0.53984 (13)	0.5665 (5)	0.0482 (11)	
H15	0.1983	0.5697	0.5562	0.058*	
C16	0.0183 (4)	0.53332 (12)	0.5676 (3)	0.0309 (8)	
C17	−0.0315 (3)	0.48885 (12)	0.5870 (3)	0.0237 (8)	
H17	−0.1278	0.4840	0.5906	0.028*	
C18	0.0611 (3)	0.45149 (12)	0.6010 (3)	0.0236 (8)	
H18	0.0260	0.4217	0.6129	0.028*	
C19	−0.0767 (4)	0.57499 (13)	0.5449 (4)	0.0369 (9)	
C20	−0.2344 (3)	0.57033 (12)	0.5405 (3)	0.0309 (8)	
H20	−0.2538	0.5439	0.5980	0.037*	
C21	−0.3046 (4)	0.61276 (17)	0.5917 (5)	0.0567 (12)	
H21A	−0.2796	0.6399	0.5413	0.068*	
H21B	−0.2693	0.6177	0.6861	0.068*	
C22	−0.4661 (4)	0.60805 (15)	0.5780 (4)	0.0438 (10)	
H22A	−0.4923	0.5835	0.6374	0.053*	
H22B	−0.5076	0.6370	0.6050	0.053*	
C23	−0.5224 (4)	0.59680 (15)	0.4343 (4)	0.0437 (10)	
H23A	−0.5036	0.6227	0.3763	0.052*	
H23B	−0.6243	0.5925	0.4277	0.052*	
C24	−0.4539 (4)	0.55266 (19)	0.3857 (4)	0.0626 (14)	
H24A	−0.4904	0.5465	0.2922	0.075*	
H24B	−0.4774	0.5262	0.4398	0.075*	
C25	−0.2919 (4)	0.55860 (17)	0.3976 (4)	0.0489 (11)	
H25A	−0.2488	0.5299	0.3705	0.059*	
H25B	−0.2682	0.5833	0.3376	0.059*	
C26	0.2923 (4)	0.33471 (14)	0.8139 (4)	0.0401 (10)	
H26A	0.2118	0.3409	0.7469	0.048*	
H26B	0.2664	0.3433	0.9021	0.048*	
C27	0.3295 (4)	0.28346 (14)	0.8125 (3)	0.0360 (9)	
C28	0.4639 (4)	0.26816 (15)	0.8651 (4)	0.0429 (10)	
H28	0.5326	0.2895	0.8993	0.052*	
C29	0.4947 (5)	0.22074 (18)	0.8663 (4)	0.0578 (12)	
H29	0.5844	0.2104	0.9010	0.069*	
C30	0.3924 (5)	0.18886 (17)	0.8161 (4)	0.0562 (12)	
H30	0.4129	0.1571	0.8187	0.067*	
C31	0.2612 (5)	0.20401 (17)	0.7627 (4)	0.0524 (12)	
H31	0.1929	0.1826	0.7274	0.063*	
C32	0.2299 (5)	0.25123 (15)	0.7612 (4)	0.0436 (10)	
H32	0.1404	0.2613	0.7249	0.052*	
C33	0.7901 (5)	0.20396 (14)	−0.3344 (4)	0.0498 (11)	
H33A	0.8514	0.2154	−0.3971	0.075*	
H33B	0.6954	0.2150	−0.3602	0.075*	
H33C	0.7906	0.1704	−0.3352	0.075*	
C34	0.8548 (4)	0.26752 (13)	−0.1822 (3)	0.0293 (8)	
C35	0.8183 (4)	0.30053 (13)	−0.2832 (3)	0.0295 (8)	
H35	0.7803	0.2912	−0.3694	0.035*	
C36	0.8395 (3)	0.34752 (12)	−0.2539 (3)	0.0263 (8)	
H36	0.8128	0.3695	−0.3209	0.032*	
C37	0.8993 (3)	0.36277 (12)	−0.1273 (3)	0.0234 (8)	
C38	0.9335 (3)	0.32901 (13)	−0.0274 (3)	0.0276 (8)	
H38	0.9725	0.3383	0.0585	0.033*	
C39	0.9106 (4)	0.28211 (13)	−0.0537 (3)	0.0326 (9)	
H39	0.9325	0.2603	0.0147	0.039*	
C40	0.9301 (3)	0.41405 (12)	−0.1030 (3)	0.0239 (8)	
H40A	1.0232	0.4211	−0.1287	0.029*	
H40B	0.8613	0.4323	−0.1605	0.029*	
C41	1.0409 (3)	0.44337 (11)	0.1179 (3)	0.0233 (8)	
C42	0.9953 (3)	0.45315 (12)	0.2555 (3)	0.0261 (8)	
H42A	0.9807	0.4863	0.2679	0.031*	
H42B	1.0655	0.4420	0.3269	0.031*	
C43	0.8571 (3)	0.42648 (11)	0.2548 (3)	0.0226 (7)	
H43	0.7922	0.4410	0.3122	0.027*	
C44	0.7968 (3)	0.42612 (11)	0.1026 (3)	0.0196 (7)	
H44	0.7486	0.3964	0.0808	0.023*	
C45	0.6947 (3)	0.46591 (11)	0.0641 (3)	0.0201 (7)	
C46	0.7387 (3)	0.50796 (12)	0.0107 (3)	0.0265 (8)	
H46	0.8334	0.5120	−0.0020	0.032*	
C47	0.6428 (3)	0.54333 (12)	−0.0232 (3)	0.0280 (8)	
H47	0.6738	0.5710	−0.0590	0.034*	
C48	0.5013 (3)	0.53856 (11)	−0.0051 (3)	0.0227 (7)	
C49	0.4561 (3)	0.49667 (11)	0.0482 (3)	0.0216 (7)	
H49	0.3614	0.4927	0.0611	0.026*	
C50	0.5530 (3)	0.46101 (12)	0.0819 (3)	0.0217 (7)	
H50	0.5220	0.4333	0.1171	0.026*	
C51	0.4028 (4)	0.57889 (12)	−0.0432 (3)	0.0281 (8)	
C52	0.2476 (3)	0.57508 (12)	−0.0231 (3)	0.0250 (8)	
H52	0.2148	0.5434	−0.0467	0.030*	
C53	0.1572 (3)	0.60977 (13)	−0.1126 (3)	0.0324 (8)	
H53A	0.1913	0.6412	−0.0924	0.039*	
H53B	0.1667	0.6034	−0.2069	0.039*	
C54	0.0015 (4)	0.60658 (13)	−0.0902 (3)	0.0352 (9)	
H54A	−0.0528	0.6293	−0.1470	0.042*	
H54B	−0.0344	0.5758	−0.1160	0.042*	
C55	−0.0171 (4)	0.61560 (13)	0.0564 (4)	0.0340 (8)	
H55A	−0.1160	0.6115	0.0694	0.041*	
H55B	0.0091	0.6476	0.0793	0.041*	
C56	0.0735 (4)	0.58269 (13)	0.1495 (3)	0.0340 (9)	
H56A	0.0381	0.5511	0.1353	0.041*	
H56B	0.0658	0.5911	0.2428	0.041*	
C57	0.2290 (4)	0.58421 (13)	0.1250 (3)	0.0307 (8)	
H57A	0.2815	0.5609	0.1812	0.037*	
H57B	0.2680	0.6146	0.1512	0.037*	
C58	0.7806 (4)	0.35197 (12)	0.3265 (3)	0.0276 (8)	
H58A	0.6996	0.3574	0.2595	0.033*	
H58B	0.7540	0.3601	0.4148	0.033*	
C59	0.8233 (3)	0.30182 (12)	0.3248 (3)	0.0255 (8)	
C60	0.9602 (4)	0.28759 (13)	0.3737 (3)	0.0298 (8)	
H60	1.0274	0.3096	0.4067	0.036*	
C61	0.9964 (4)	0.24096 (13)	0.3735 (3)	0.0356 (9)	
H61	1.0882	0.2319	0.4056	0.043*	
C62	0.8979 (4)	0.20761 (14)	0.3260 (3)	0.0384 (10)	
H62	0.9228	0.1762	0.3275	0.046*	
C63	0.7621 (4)	0.22115 (14)	0.2763 (4)	0.0380 (9)	
H63	0.6952	0.1989	0.2441	0.046*	
C64	0.7261 (4)	0.26768 (13)	0.2747 (3)	0.0325 (9)	
H64	0.6350	0.2766	0.2395	0.039*	
O1	0.3309 (3)	0.21905 (8)	0.2685 (2)	0.0342 (6)	
O2	0.6627 (2)	0.43771 (8)	0.5647 (2)	0.0298 (6)	
O3	0.4116 (2)	0.36182 (9)	0.7845 (2)	0.0319 (6)	
O4	−0.0242 (3)	0.61327 (10)	0.5212 (3)	0.0619 (9)	
O5	0.8393 (3)	0.22030 (9)	−0.2012 (2)	0.0390 (6)	
O6	1.1596 (2)	0.44863 (8)	0.0834 (2)	0.0326 (6)	
O7	0.8971 (2)	0.38020 (8)	0.2972 (2)	0.0258 (5)	
N1	0.4250 (2)	0.42119 (10)	0.5347 (2)	0.0215 (6)	
N2	0.9258 (3)	0.42805 (10)	0.0370 (2)	0.0219 (6)	
O8	0.4500 (3)	0.61465 (9)	−0.0869 (3)	0.0412 (6)	

### Atomic displacement parameters (Å^2^)

**Table:**

	*U*^11^	*U*^22^	*U*^33^	*U*^12^	*U*^13^	*U*^23^
C1	0.064 (3)	0.031 (2)	0.038 (2)	−0.011 (2)	0.007 (2)	−0.0052 (17)
C2	0.0277 (19)	0.023 (2)	0.0313 (19)	−0.0009 (15)	0.0109 (15)	0.0013 (16)
C3	0.0307 (19)	0.032 (2)	0.0224 (17)	−0.0040 (16)	0.0043 (14)	−0.0008 (16)
C4	0.0244 (18)	0.028 (2)	0.0238 (17)	−0.0021 (15)	0.0049 (14)	0.0022 (15)
C5	0.0199 (16)	0.0258 (19)	0.0166 (15)	0.0004 (14)	0.0062 (13)	−0.0025 (14)
C6	0.0282 (19)	0.032 (2)	0.0186 (16)	0.0002 (16)	0.0032 (14)	0.0003 (15)
C7	0.033 (2)	0.031 (2)	0.0252 (18)	0.0024 (17)	0.0063 (15)	0.0082 (16)
C8	0.0213 (17)	0.030 (2)	0.0144 (15)	−0.0006 (14)	0.0032 (13)	−0.0007 (13)
C9	0.0220 (18)	0.025 (2)	0.0278 (17)	−0.0039 (15)	0.0017 (15)	−0.0031 (15)
C10	0.0294 (19)	0.048 (2)	0.0234 (17)	−0.0061 (18)	0.0014 (15)	−0.0053 (17)
C11	0.0260 (18)	0.040 (2)	0.0195 (17)	−0.0036 (16)	0.0062 (14)	−0.0041 (15)
C12	0.0205 (17)	0.032 (2)	0.0245 (17)	−0.0038 (15)	0.0076 (14)	−0.0041 (15)
C13	0.0233 (18)	0.032 (2)	0.0201 (16)	−0.0046 (15)	0.0044 (13)	−0.0098 (15)
C14	0.021 (2)	0.035 (2)	0.077 (3)	−0.0073 (18)	0.0156 (19)	−0.011 (2)
C15	0.033 (2)	0.024 (2)	0.091 (3)	−0.0079 (18)	0.019 (2)	−0.009 (2)
C16	0.030 (2)	0.027 (2)	0.0366 (19)	−0.0041 (16)	0.0077 (16)	−0.0095 (16)
C17	0.0194 (17)	0.032 (2)	0.0195 (16)	−0.0028 (16)	0.0030 (13)	−0.0028 (15)
C18	0.0247 (18)	0.031 (2)	0.0160 (15)	−0.0066 (16)	0.0035 (13)	0.0018 (14)
C19	0.038 (2)	0.025 (2)	0.048 (2)	−0.0058 (18)	0.0065 (18)	−0.0083 (18)
C20	0.0284 (19)	0.025 (2)	0.0386 (19)	0.0005 (15)	0.0017 (16)	−0.0051 (16)
C21	0.045 (2)	0.055 (3)	0.069 (3)	0.002 (2)	0.002 (2)	−0.035 (2)
C22	0.043 (2)	0.044 (3)	0.046 (2)	0.004 (2)	0.0114 (18)	−0.014 (2)
C23	0.037 (2)	0.060 (3)	0.034 (2)	0.014 (2)	0.0033 (17)	0.0076 (19)
C24	0.047 (3)	0.100 (4)	0.038 (2)	0.017 (3)	−0.005 (2)	−0.026 (2)
C25	0.044 (2)	0.069 (3)	0.033 (2)	0.019 (2)	0.0019 (18)	−0.009 (2)
C26	0.033 (2)	0.053 (3)	0.037 (2)	−0.0020 (19)	0.0148 (17)	0.0120 (19)
C27	0.038 (2)	0.047 (2)	0.0262 (18)	0.002 (2)	0.0161 (16)	0.0091 (17)
C28	0.033 (2)	0.045 (3)	0.054 (2)	0.0050 (19)	0.0184 (19)	0.008 (2)
C29	0.050 (3)	0.070 (4)	0.059 (3)	0.013 (3)	0.027 (2)	0.007 (3)
C30	0.071 (3)	0.049 (3)	0.054 (3)	0.006 (3)	0.032 (2)	−0.006 (2)
C31	0.064 (3)	0.055 (3)	0.041 (2)	−0.015 (2)	0.020 (2)	−0.006 (2)
C32	0.046 (3)	0.054 (3)	0.033 (2)	0.000 (2)	0.0133 (18)	0.0033 (19)
C33	0.077 (3)	0.036 (2)	0.039 (2)	−0.006 (2)	0.013 (2)	−0.0042 (19)
C34	0.032 (2)	0.027 (2)	0.0318 (19)	0.0035 (16)	0.0149 (16)	−0.0018 (16)
C35	0.033 (2)	0.036 (2)	0.0206 (17)	−0.0036 (17)	0.0075 (15)	−0.0034 (16)
C36	0.0263 (18)	0.036 (2)	0.0178 (16)	0.0048 (16)	0.0067 (14)	0.0059 (16)
C37	0.0210 (17)	0.031 (2)	0.0194 (16)	0.0019 (15)	0.0079 (14)	0.0018 (14)
C38	0.0272 (19)	0.037 (2)	0.0189 (17)	0.0048 (16)	0.0053 (14)	−0.0001 (16)
C39	0.036 (2)	0.037 (2)	0.0251 (18)	0.0073 (18)	0.0050 (16)	0.0050 (16)
C40	0.0237 (17)	0.033 (2)	0.0163 (15)	0.0026 (15)	0.0064 (13)	0.0037 (14)
C41	0.0233 (19)	0.0219 (18)	0.0241 (17)	−0.0001 (15)	−0.0002 (15)	0.0101 (14)
C42	0.0272 (18)	0.030 (2)	0.0213 (16)	−0.0034 (16)	0.0011 (14)	0.0006 (14)
C43	0.0299 (18)	0.0212 (19)	0.0173 (15)	0.0028 (15)	0.0051 (13)	0.0012 (14)
C44	0.0189 (16)	0.0198 (18)	0.0212 (16)	−0.0012 (14)	0.0072 (13)	0.0018 (14)
C45	0.0196 (17)	0.0259 (19)	0.0151 (14)	−0.0031 (14)	0.0038 (13)	−0.0055 (14)
C46	0.0193 (18)	0.028 (2)	0.0315 (18)	−0.0035 (15)	0.0003 (14)	0.0010 (15)
C47	0.0296 (19)	0.027 (2)	0.0275 (17)	−0.0059 (16)	0.0042 (15)	0.0019 (15)
C48	0.0239 (18)	0.0206 (18)	0.0234 (16)	−0.0007 (14)	0.0024 (13)	−0.0003 (14)
C49	0.0207 (17)	0.027 (2)	0.0173 (15)	−0.0005 (15)	0.0040 (13)	−0.0028 (14)
C50	0.0230 (18)	0.0272 (19)	0.0156 (15)	−0.0046 (15)	0.0052 (13)	−0.0013 (14)
C51	0.034 (2)	0.024 (2)	0.0268 (18)	−0.0029 (17)	0.0055 (15)	−0.0021 (15)
C52	0.0243 (18)	0.0178 (18)	0.0325 (18)	0.0011 (15)	0.0019 (15)	0.0008 (15)
C53	0.0332 (19)	0.032 (2)	0.0314 (18)	0.0024 (17)	0.0019 (16)	0.0038 (16)
C54	0.032 (2)	0.027 (2)	0.044 (2)	0.0041 (17)	−0.0043 (17)	−0.0009 (18)
C55	0.0286 (19)	0.024 (2)	0.051 (2)	−0.0002 (16)	0.0080 (16)	−0.0042 (17)
C56	0.032 (2)	0.037 (2)	0.0346 (19)	0.0016 (17)	0.0099 (16)	−0.0052 (16)
C57	0.0285 (19)	0.036 (2)	0.0274 (18)	0.0003 (16)	0.0006 (15)	0.0007 (15)
C58	0.0299 (19)	0.029 (2)	0.0253 (17)	0.0004 (16)	0.0100 (15)	0.0035 (15)
C59	0.0286 (18)	0.030 (2)	0.0195 (16)	0.0001 (16)	0.0108 (14)	0.0044 (15)
C60	0.035 (2)	0.028 (2)	0.0286 (18)	0.0009 (17)	0.0104 (15)	−0.0003 (16)
C61	0.032 (2)	0.040 (2)	0.036 (2)	0.0064 (18)	0.0103 (16)	0.0043 (17)
C62	0.050 (3)	0.030 (2)	0.037 (2)	0.0064 (19)	0.0125 (19)	−0.0038 (17)
C63	0.041 (2)	0.034 (2)	0.041 (2)	−0.0049 (19)	0.0116 (18)	−0.0035 (18)
C64	0.030 (2)	0.038 (2)	0.0308 (19)	−0.0014 (18)	0.0096 (16)	−0.0017 (17)
O1	0.0473 (16)	0.0222 (14)	0.0343 (13)	−0.0019 (11)	0.0095 (11)	−0.0026 (11)
O2	0.0232 (13)	0.0340 (15)	0.0327 (13)	−0.0076 (11)	0.0062 (10)	0.0014 (11)
O3	0.0292 (13)	0.0431 (17)	0.0244 (12)	0.0000 (12)	0.0070 (10)	0.0093 (11)
O4	0.0408 (17)	0.0285 (17)	0.117 (3)	−0.0060 (14)	0.0120 (16)	−0.0074 (17)
O5	0.0519 (17)	0.0327 (16)	0.0340 (13)	0.0044 (13)	0.0111 (12)	−0.0021 (12)
O6	0.0226 (13)	0.0391 (15)	0.0368 (14)	−0.0047 (11)	0.0069 (11)	0.0110 (11)
O7	0.0250 (12)	0.0279 (14)	0.0254 (12)	−0.0007 (10)	0.0067 (10)	0.0067 (10)
N1	0.0167 (13)	0.0323 (16)	0.0162 (12)	−0.0066 (12)	0.0052 (11)	−0.0039 (12)
N2	0.0184 (14)	0.0318 (17)	0.0163 (12)	−0.0017 (12)	0.0049 (11)	0.0012 (12)
O8	0.0370 (15)	0.0243 (15)	0.0645 (17)	0.0000 (12)	0.0147 (12)	0.0103 (13)

### Geometric parameters (Å, º)

**Table:**

C1—O1	1.425 (4)	C33—O5	1.432 (4)
C1—H1A	0.9600	C33—H33A	0.9600
C1—H1B	0.9600	C33—H33B	0.9600
C1—H1C	0.9600	C33—H33C	0.9600
C2—O1	1.368 (4)	C34—O5	1.372 (4)
C2—C7	1.385 (5)	C34—C39	1.390 (5)
C2—C3	1.397 (5)	C34—C35	1.394 (5)
C3—C4	1.391 (5)	C35—C36	1.387 (5)
C3—H3	0.9300	C35—H35	0.9300
C4—C5	1.383 (4)	C36—C37	1.390 (4)
C4—H4	0.9300	C36—H36	0.9300
C5—C6	1.406 (5)	C37—C38	1.398 (5)
C5—C8	1.508 (5)	C37—C40	1.512 (5)
C6—C7	1.375 (5)	C38—C39	1.381 (5)
C6—H6	0.9300	C38—H38	0.9300
C7—H7	0.9300	C39—H39	0.9300
C8—N1	1.456 (4)	C40—N2	1.455 (4)
C8—H8A	0.9700	C40—H40A	0.9700
C8—H8B	0.9700	C40—H40B	0.9700
C9—O2	1.226 (4)	C41—O6	1.224 (4)
C9—N1	1.349 (4)	C41—N2	1.352 (4)
C9—C10	1.514 (4)	C41—C42	1.511 (4)
C10—C11	1.514 (5)	C42—C43	1.518 (4)
C10—H10A	0.9700	C42—H42A	0.9700
C10—H10B	0.9700	C42—H42B	0.9700
C11—O3	1.420 (4)	C43—O7	1.429 (4)
C11—C12	1.559 (4)	C43—C44	1.556 (4)
C11—H11	0.9800	C43—H43	0.9800
C12—N1	1.461 (4)	C44—N2	1.455 (4)
C12—C13	1.504 (5)	C44—C45	1.517 (4)
C12—H12	0.9800	C44—H44	0.9800
C13—C14	1.380 (5)	C45—C50	1.385 (4)
C13—C18	1.390 (5)	C45—C46	1.400 (5)
C14—C15	1.377 (5)	C46—C47	1.378 (5)
C14—H14	0.9300	C46—H46	0.9300
C15—C16	1.388 (5)	C47—C48	1.383 (4)
C15—H15	0.9300	C47—H47	0.9300
C16—C17	1.380 (5)	C48—C49	1.400 (5)
C16—C19	1.497 (5)	C48—C51	1.507 (5)
C17—C18	1.382 (5)	C49—C50	1.389 (5)
C17—H17	0.9300	C49—H49	0.9300
C18—H18	0.9300	C50—H50	0.9300
C19—O4	1.239 (5)	C51—O8	1.219 (4)
C19—C20	1.499 (5)	C51—C52	1.515 (5)
C20—C25	1.500 (5)	C52—C57	1.526 (5)
C20—C21	1.504 (5)	C52—C53	1.529 (4)
C20—H20	0.9800	C52—H52	0.9800
C21—C22	1.529 (5)	C53—C54	1.524 (5)
C21—H21A	0.9700	C53—H53A	0.9700
C21—H21B	0.9700	C53—H53B	0.9700
C22—C23	1.502 (5)	C54—C55	1.513 (5)
C22—H22A	0.9700	C54—H54A	0.9700
C22—H22B	0.9700	C54—H54B	0.9700
C23—C24	1.526 (6)	C55—C56	1.517 (5)
C23—H23A	0.9700	C55—H55A	0.9700
C23—H23B	0.9700	C55—H55B	0.9700
C24—C25	1.539 (6)	C56—C57	1.525 (5)
C24—H24A	0.9700	C56—H56A	0.9700
C24—H24B	0.9700	C56—H56B	0.9700
C25—H25A	0.9700	C57—H57A	0.9700
C25—H25B	0.9700	C57—H57B	0.9700
C26—O3	1.432 (4)	C58—O7	1.428 (4)
C26—C27	1.511 (6)	C58—C59	1.494 (5)
C26—H26A	0.9700	C58—H58A	0.9700
C26—H26B	0.9700	C58—H58B	0.9700
C27—C32	1.376 (5)	C59—C60	1.395 (5)
C27—C28	1.392 (5)	C59—C64	1.396 (5)
C28—C29	1.389 (6)	C60—C61	1.379 (5)
C28—H28	0.9300	C60—H60	0.9300
C29—C30	1.383 (6)	C61—C62	1.381 (5)
C29—H29	0.9300	C61—H61	0.9300
C30—C31	1.367 (6)	C62—C63	1.382 (5)
C30—H30	0.9300	C62—H62	0.9300
C31—C32	1.385 (6)	C63—C64	1.376 (5)
C31—H31	0.9300	C63—H63	0.9300
C32—H32	0.9300	C64—H64	0.9300
			
O1—C1—H1A	109.5	H33B—C33—H33C	109.5
O1—C1—H1B	109.5	O5—C34—C39	116.5 (3)
H1A—C1—H1B	109.5	O5—C34—C35	123.8 (3)
O1—C1—H1C	109.5	C39—C34—C35	119.7 (3)
H1A—C1—H1C	109.5	C36—C35—C34	119.3 (3)
H1B—C1—H1C	109.5	C36—C35—H35	120.4
O1—C2—C7	116.1 (3)	C34—C35—H35	120.4
O1—C2—C3	124.6 (3)	C35—C36—C37	122.0 (3)
C7—C2—C3	119.3 (3)	C35—C36—H36	119.0
C4—C3—C2	119.2 (3)	C37—C36—H36	119.0
C4—C3—H3	120.4	C36—C37—C38	117.6 (3)
C2—C3—H3	120.4	C36—C37—C40	120.1 (3)
C5—C4—C3	122.1 (3)	C38—C37—C40	122.3 (3)
C5—C4—H4	118.9	C39—C38—C37	121.3 (3)
C3—C4—H4	118.9	C39—C38—H38	119.3
C4—C5—C6	117.5 (3)	C37—C38—H38	119.3
C4—C5—C8	120.5 (3)	C38—C39—C34	120.1 (3)
C6—C5—C8	122.0 (3)	C38—C39—H39	120.0
C7—C6—C5	121.0 (3)	C34—C39—H39	120.0
C7—C6—H6	119.5	N2—C40—C37	113.2 (3)
C5—C6—H6	119.5	N2—C40—H40A	108.9
C6—C7—C2	120.7 (3)	C37—C40—H40A	108.9
C6—C7—H7	119.6	N2—C40—H40B	108.9
C2—C7—H7	119.6	C37—C40—H40B	108.9
N1—C8—C5	112.4 (3)	H40A—C40—H40B	107.7
N1—C8—H8A	109.1	O6—C41—N2	125.4 (3)
C5—C8—H8A	109.1	O6—C41—C42	127.0 (3)
N1—C8—H8B	109.1	N2—C41—C42	107.6 (3)
C5—C8—H8B	109.1	C41—C42—C43	104.0 (2)
H8A—C8—H8B	107.8	C41—C42—H42A	111.0
O2—C9—N1	125.7 (3)	C43—C42—H42A	111.0
O2—C9—C10	126.9 (3)	C41—C42—H42B	111.0
N1—C9—C10	107.5 (3)	C43—C42—H42B	111.0
C9—C10—C11	104.7 (3)	H42A—C42—H42B	109.0
C9—C10—H10A	110.8	O7—C43—C42	105.4 (2)
C11—C10—H10A	110.8	O7—C43—C44	109.6 (2)
C9—C10—H10B	110.8	C42—C43—C44	103.1 (2)
C11—C10—H10B	110.8	O7—C43—H43	112.7
H10A—C10—H10B	108.9	C42—C43—H43	112.7
O3—C11—C10	106.8 (3)	C44—C43—H43	112.7
O3—C11—C12	110.4 (3)	N2—C44—C45	113.9 (3)
C10—C11—C12	103.2 (3)	N2—C44—C43	101.7 (2)
O3—C11—H11	112.0	C45—C44—C43	113.1 (2)
C10—C11—H11	112.0	N2—C44—H44	109.3
C12—C11—H11	112.0	C45—C44—H44	109.3
N1—C12—C13	113.1 (3)	C43—C44—H44	109.3
N1—C12—C11	102.0 (2)	C50—C45—C46	118.3 (3)
C13—C12—C11	114.2 (3)	C50—C45—C44	119.6 (3)
N1—C12—H12	109.1	C46—C45—C44	122.1 (3)
C13—C12—H12	109.1	C47—C46—C45	120.5 (3)
C11—C12—H12	109.1	C47—C46—H46	119.8
C14—C13—C18	118.0 (3)	C45—C46—H46	119.8
C14—C13—C12	121.7 (3)	C46—C47—C48	121.3 (3)
C18—C13—C12	120.4 (3)	C46—C47—H47	119.3
C15—C14—C13	121.2 (3)	C48—C47—H47	119.3
C15—C14—H14	119.4	C47—C48—C49	118.7 (3)
C13—C14—H14	119.4	C47—C48—C51	118.4 (3)
C14—C15—C16	120.6 (4)	C49—C48—C51	122.9 (3)
C14—C15—H15	119.7	C50—C49—C48	119.8 (3)
C16—C15—H15	119.7	C50—C49—H49	120.1
C17—C16—C15	118.7 (3)	C48—C49—H49	120.1
C17—C16—C19	123.2 (3)	C45—C50—C49	121.4 (3)
C15—C16—C19	118.1 (3)	C45—C50—H50	119.3
C16—C17—C18	120.3 (3)	C49—C50—H50	119.3
C16—C17—H17	119.9	O8—C51—C48	119.4 (3)
C18—C17—H17	119.9	O8—C51—C52	120.8 (3)
C17—C18—C13	121.2 (3)	C48—C51—C52	119.8 (3)
C17—C18—H18	119.4	C51—C52—C57	109.9 (2)
C13—C18—H18	119.4	C51—C52—C53	111.4 (3)
O4—C19—C16	119.1 (3)	C57—C52—C53	109.3 (3)
O4—C19—C20	119.7 (3)	C51—C52—H52	108.8
C16—C19—C20	121.0 (3)	C57—C52—H52	108.8
C19—C20—C25	107.4 (3)	C53—C52—H52	108.8
C19—C20—C21	113.5 (3)	C54—C53—C52	111.3 (3)
C25—C20—C21	112.1 (3)	C54—C53—H53A	109.4
C19—C20—H20	107.9	C52—C53—H53A	109.4
C25—C20—H20	107.9	C54—C53—H53B	109.4
C21—C20—H20	107.9	C52—C53—H53B	109.4
C20—C21—C22	112.2 (3)	H53A—C53—H53B	108.0
C20—C21—H21A	109.2	C55—C54—C53	110.9 (3)
C22—C21—H21A	109.2	C55—C54—H54A	109.5
C20—C21—H21B	109.2	C53—C54—H54A	109.5
C22—C21—H21B	109.2	C55—C54—H54B	109.5
H21A—C21—H21B	107.9	C53—C54—H54B	109.5
C23—C22—C21	110.2 (3)	H54A—C54—H54B	108.1
C23—C22—H22A	109.6	C54—C55—C56	111.1 (3)
C21—C22—H22A	109.6	C54—C55—H55A	109.4
C23—C22—H22B	109.6	C56—C55—H55A	109.4
C21—C22—H22B	109.6	C54—C55—H55B	109.4
H22A—C22—H22B	108.1	C56—C55—H55B	109.4
C22—C23—C24	111.2 (3)	H55A—C55—H55B	108.0
C22—C23—H23A	109.4	C55—C56—C57	112.0 (3)
C24—C23—H23A	109.4	C55—C56—H56A	109.2
C22—C23—H23B	109.4	C57—C56—H56A	109.2
C24—C23—H23B	109.4	C55—C56—H56B	109.2
H23A—C23—H23B	108.0	C57—C56—H56B	109.2
C23—C24—C25	110.1 (4)	H56A—C56—H56B	107.9
C23—C24—H24A	109.6	C56—C57—C52	111.9 (3)
C25—C24—H24A	109.6	C56—C57—H57A	109.2
C23—C24—H24B	109.6	C52—C57—H57A	109.2
C25—C24—H24B	109.6	C56—C57—H57B	109.2
H24A—C24—H24B	108.2	C52—C57—H57B	109.2
C20—C25—C24	110.3 (3)	H57A—C57—H57B	107.9
C20—C25—H25A	109.6	O7—C58—C59	108.9 (3)
C24—C25—H25A	109.6	O7—C58—H58A	109.9
C20—C25—H25B	109.6	C59—C58—H58A	109.9
C24—C25—H25B	109.6	O7—C58—H58B	109.9
H25A—C25—H25B	108.1	C59—C58—H58B	109.9
O3—C26—C27	109.5 (3)	H58A—C58—H58B	108.3
O3—C26—H26A	109.8	C60—C59—C64	118.0 (3)
C27—C26—H26A	109.8	C60—C59—C58	121.4 (3)
O3—C26—H26B	109.8	C64—C59—C58	120.6 (3)
C27—C26—H26B	109.8	C61—C60—C59	120.3 (3)
H26A—C26—H26B	108.2	C61—C60—H60	119.8
C32—C27—C28	119.2 (4)	C59—C60—H60	119.8
C32—C27—C26	120.3 (3)	C60—C61—C62	120.8 (3)
C28—C27—C26	120.5 (4)	C60—C61—H61	119.6
C29—C28—C27	119.6 (4)	C62—C61—H61	119.6
C29—C28—H28	120.2	C61—C62—C63	119.6 (4)
C27—C28—H28	120.2	C61—C62—H62	120.2
C30—C29—C28	120.3 (4)	C63—C62—H62	120.2
C30—C29—H29	119.9	C64—C63—C62	119.8 (4)
C28—C29—H29	119.9	C64—C63—H63	120.1
C31—C30—C29	120.0 (5)	C62—C63—H63	120.1
C31—C30—H30	120.0	C63—C64—C59	121.5 (3)
C29—C30—H30	120.0	C63—C64—H64	119.3
C30—C31—C32	119.9 (4)	C59—C64—H64	119.3
C30—C31—H31	120.0	C2—O1—C1	117.1 (3)
C32—C31—H31	120.0	C11—O3—C26	114.1 (3)
C27—C32—C31	121.0 (4)	C34—O5—C33	117.9 (3)
C27—C32—H32	119.5	C58—O7—C43	113.6 (2)
C31—C32—H32	119.5	C9—N1—C8	123.1 (3)
O5—C33—H33A	109.5	C9—N1—C12	114.3 (2)
O5—C33—H33B	109.5	C8—N1—C12	122.3 (2)
H33A—C33—H33B	109.5	C41—N2—C44	114.2 (2)
O5—C33—H33C	109.5	C41—N2—C40	123.2 (3)
H33A—C33—H33C	109.5	C44—N2—C40	122.6 (2)

### Hydrogen-bond geometry (Å, º)

**Table:**

*D*—H···*A*	*D*—H	H···*A*	*D*···*A*	*D*—H···*A*
C62—H62···O4^i^	0.93	2.47	3.260 (5)	143
C17—H17···O2^ii^	0.93	2.38	3.237 (4)	153
C49—H49···O6^ii^	0.93	2.33	3.190 (4)	154

Symmetry codes: (i) −*x*+1, *y*−1/2, −*z*+1; (ii) *x*−1, *y*, *z*.
